# Involving multiple stakeholders in assessing and reviewing a novel data visualisation tool for a national neonatal data asset

**DOI:** 10.1136/bmjhci-2022-100694

**Published:** 2023-01-31

**Authors:** William Bishop Lammons, Becky Moss, Charlie Bignell, Chris Gale, Adam MacBride, Ricardo Ribas, Cheryl Battersby, Neena Modi

**Affiliations:** 1 Department of Applied Health Research, UCL, London, UK; 2 Section of Neonatal Medicine, School of Primary Care and Public Health, Imperial College London, London, UK; 3 Public co-author, n/a, London, UK

**Keywords:** patient involvement, data visualization, electronic health records, health information systems

## Abstract

**Objectives:**

We involved public and professional stakeholders to assess a novel data interrogation tool, the Neonatal Health Intelligence Tool, for a National Data Asset, the National Neonatal Research Database.

**Methods:**

We recruited parents, preterm adults, data managers, clinicians, network managers and researchers (trialists and epidemiologists) for consultations demonstrating a prototype tool and semi-structured discussion. A thematic analysis of consultations is reported by stakeholder group.

**Results:**

We held nine on-line consultations (March–December 2021), with 24 stakeholders: parents (n=8), preterm adults (n=2), data managers (n=3), clinicians (n=3), network managers (n=2), triallists (n=3) and epidemiologists (n=3). We identified four themes from parents/preterm adults: struggling to consume information, Dads and data, bring data to life and yearning for predictions; five themes from data managers/clinicians/network managers: benchmarking, clinical outcomes, transfers and activity, the impact of socioeconomic background and ethnicity, and timeliness of updates and widening availability; and one theme from researchers: interrogating the data.

**Discussion:**

Other patient and public involvement (PPI) studies have reported that data tools generate concerns; our stakeholders had none. They were unanimously supportive and enthusiastic, citing visualisation as the tool’s greatest strength. Stakeholders had no criticisms; instead, they recognised the tool’s potential and wanted more features. Parents saw the tool as an opportunity to inform themselves without burdening clinicians, while clinicians welcomed an aid to explaining potential outcomes to parents.

**Conclusion:**

All stakeholder groups recognised the need for the tool, praising its content and format. PPI consultations with all key groups, and their synthesis, illustrated desire for additional uses from it.

**WHAT IS ALREADY KNOWN ON THIS TOPIC**

- Patient and public involvement provides benefits across all aspects of health and social care research and should be the gold standard from study inception to dissemination and evaluation.
- Data interrogation tools allow for analysis of large-scale point-of-care data on a nationwide scale.

**WHAT THIS STUDY ADDS**

- This study demonstrates the importance of extending standard patient and public involvement to include a wider range of stakeholders and synthesising the contributions of each group to maximise the uses and value of data interrogation tools.
- This study illustrates how qualitative analysis can identify connections across differing stakeholder groups in the context of a public involvement activity.

**HOW THIS STUDY MIGHT AFFECT RESEARCH, PRACTICE OR POLICY**

- An effective and useful data interrogation tool will encourage national and international researchers to use the health data contained in the National Neonatal Research Database.
- Clinicians will be able to use the tool in their practice as an aid to explaining neonatal outcomes to parents.
- Interrogation of the data set will enable policy makers to explore influences on neonatal outcomes.

## Introduction

### The National Neonatal Research Database

The National Neonatal Research Database (NNRD) is a data asset containing detailed clinical information, a standard extract from the Electronic Patient Records of admissions to all NHS neonatal units in England, Wales, Scotland and the Isle of Man.^1 2^ The extract (the Neonatal Data Set, an approved NHS Information Standard) undergoes quality assurance prior to deposition in the NNRD. The NNRD is a UK Research Ethics Service–approved database (10/80803/151) and supports national and international neonatal research. Access is through the Health Data Research UK Gateway (https://www.imperial.ac.uk/neonatal-data-analysis-unit/neonatal-data-analysis-unit/utilising-the-national-neonatal-research-database/).

**Figure 1:**
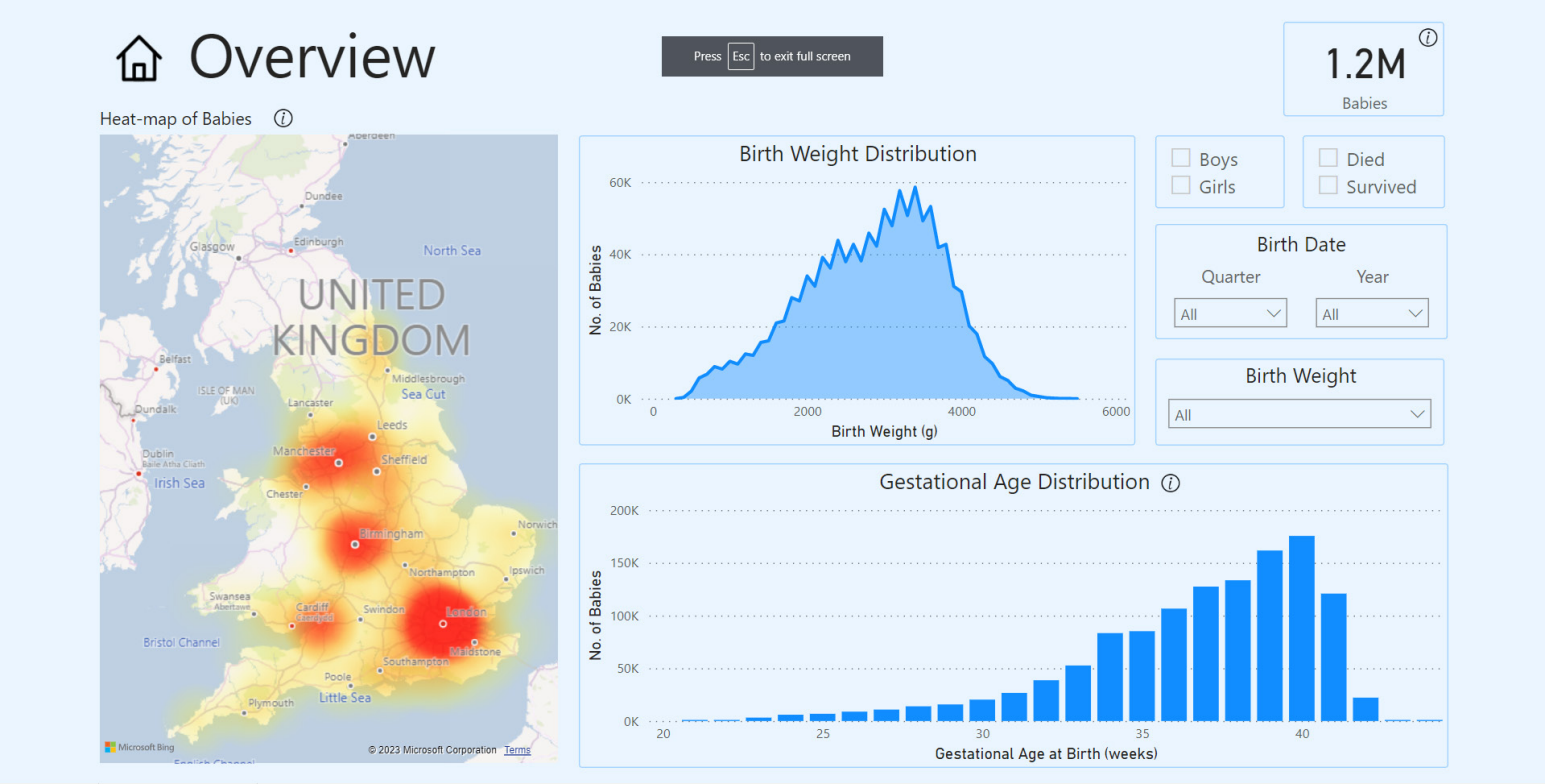
Neonatal Health Intelligence Tool, Overview feature showing birth weight, gestational age trends across the UK.

**Figure 2:**
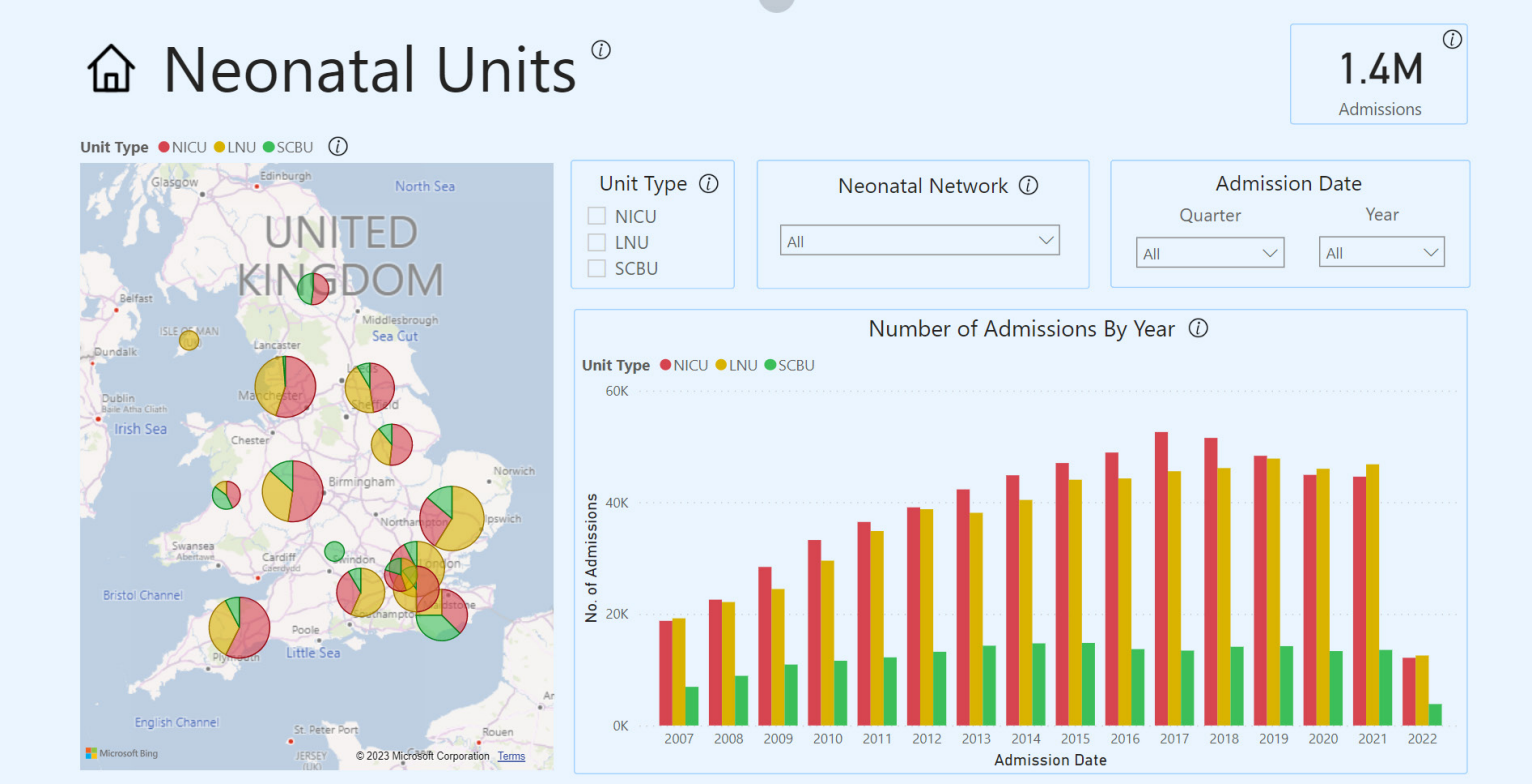
Neonatal Health Intelligence Tool, Survival feature showing rates of survival against birth weight and gestational age variables.

In 2020, with the support from Medical Research Council, we began developing a Neonatal Health Intelligence Tool (figures 1 and 2; https://www.imperial.ac.uk/neonatal-data-analysis-unit/neonatal-data-analysis-unit/neonatal-data-visualisations/) that enables interrogation of the NNRD^3^ and viewing of data on babies requiring neonatal care from 2008 by neonatal network. The tool is an online web-based application using graphs and charts that show data and trends on neonatal outcomes, such as necrotising enterocolitis and bronchopulmonary dysplasia, in a visual, easy-to-use format.

### Parent, patient and public involvement

Commonly termed ‘Patient and Public Involvement’ (PPI), neonatal medicine uses ‘Parent, Patient and Public Involvement’ (PPPI), which focuses on collaborating with parents, patients and members of the public to ensure those impacted by studies contribute to their design and delivery.^4–6^ Some PPPI work combines the voices of parents, patients and the public with those of clinical or research staff,^7–9^ but these consultations are commonly reported discretely by individual groups: the public, clinicians or researchers.^10 11^ These consultations’ outputs are also typically patient-focused communication materials or decision-making tools, rather than data visualisation tools.

National Institute of Health and Care Research (NIHR) standards of public involvement focus on patients and the public as service users, without considering clinician or researcher stakeholder roles.^12^ However, since the NNRD data tool benefits a wide range of stakeholders, we applied the concepts and methods of public and patient consultations to an exhaustive list of professional stakeholders who would use these tools to explore neonatal activity and outcomes. We therefore term this approach ‘public and stakeholder involvement,’ a technique which has received support^9^ but has been applied to few data visualisation tools.

### Study objectives

Our objectives for these stakeholder consultations were to:

- Understand what all stakeholder groups need from the tool
- Identify functionality issues
- Optimise the efficacy of the design process by synthesising all stakeholder views

## Methods

### Recruitment

We recruited parents and adults who were born preterm (‘preterm adults’) through an existing network of individuals (neoWONDER; https://www.neowonder.org.uk/) who consented to be invited to participate in research by email,^13^ and professionals through our contacts and NHS network lists, also by email.

### Consultation structure

We combined PPPI with qualitative methodologies to learn about each stakeholder group’s unique needs.^14 15^ Each consultation was attended by one to four participants; parent and preterm adults were mixed groups, and the remaining stakeholders were recruited to attend groups based on their specific requirements of the NNRD. Each session featured:

1. A demonstration of the NNRD Neonatal Health Intelligence Tool’s features led by their designer (CB), lasting approximately 15 min (figures 1 and 2)
2. Stakeholder question-and-answer session in response to the demonstration.
3. Semi-structured focus group discussion (individual interviews, n=2, used the same topic guide)

Sessions were held by video Zoom meeting. Participant video was on by default, but to support inclusivity, participants were encouraged to have video on or off at their discretion. Session lengths averaged 75 min and were video recorded with verbal consent. Zoom’s closed captioning function was used to create a raw transcript which was saved and then edited by the authors for clarity. The focus group topic guide incorporated deductive questions (eg, ‘*would this be useful?*’) and inductive questions (eg, *‘why would this be useful?*’), to allow further ideas to emerge from discussion.^15^ A complete list of topic guide questions is included in online supplemental appendix 1. The semi-structured format allowed flexibility to probe topics particular to the discussion and the stakeholder audience.^16^ The social context of sharing and discussing ideas in a focus group format also allowed for ‘reflection and refinement’ of participants’ ideas, which can “deepen respondents’ insights into their own circumstances, attitudes or behaviour”.^17^ We offered participants the chance to contribute through the chat function, speak in an individual interview and called participants individually during meetings to ensure all had a chance to verbalise their opinions. We stopped recruiting when we ceased to obtain further insights; this is referred to in qualitative methodology as data saturation.^18^ Professional consultations were chaired by a neonatologist (CG) and parent/preterm adult consultations by the PPPI Leads (BM, WL).

10.1136/bmjhci-2022-100694.supp1 Supplementary data

### Analysis

We combine approaches for understanding relationships between users and digital tools, reporting thematic analysis of consultations by stakeholder group.^17 19^ BM and WBL analysed transcripts by individual stakeholder group using each using a combination of NVivo V.1.3, NVivo V.16 (QSR International) and Microsoft Word. This revealed the unique needs of each, while highlighting commonalities between them. We reviewed transcripts on an individual stakeholder group basis, then created thematic codes to identify key subthemes.^20^ WL then conducted a final iterative analysis comparing and synthesising themes across differing stakeholder groups. BM and WBL reviewed one another’s analyses throughout this process to ensure themes were comprehensively captured.^21^ We report key PPI checklist items in the Guidance for Reporting Involvement of Patients and the Public 2 (GRIPP2, online supplemental appendix 2).^22^

10.1136/bmjhci-2022-100694.supp2 Supplementary data

## Results

We held nine digital consultation groups between March and December 2021, with 24 stakeholders: parents (n=8), preterm adults (n=2), data managers (n=3), clinicians (n=3), network managers (n=2), triallists (n=3), and epidemiologists (n=3).

### Parents and preterm adults

The tool was enthusiastically received by parents (Mums=6, Dads=2) and preterm adults and described as something they would use. The visual nature was regarded as helpful and easy to grasp. Four salient themes were identified: struggling to consume information, Dads and data, bringing the data to life and yearning for predictions.

#### Struggling to consume information

Parents described difficulty with comprehending health information due to the emotional strain of their baby being on the neonatal unit. Indeed, one felt the amount of data featured in the tool was excessive:

‘*That is way too much data… I think I would sit down… look at that… and say, “Oh my god”, and… panic’* (Mum).

However, while another participant concurred that the tool contained a large amount of information, they indicated that some parents desired even more than this. They described a complex balance between the volume of data required for decision-making and the type or amount of information capable of worsening their distress, particularly when the baby’s survival is precarious:

*‘…We had [him at] 23 [weeks]… [the doctors] said: “Baby is going to be born. We can either take them away from you for… assessment, but by the time we hand him back to you, he might already be dead. Or you can give him a cuddle as he passes away. We’ll come back in 30 minutes…” It is horrific. A decision needs to be made, ultimately, so you need… to know these things…’* (Mum).

#### Dads and data

Across all parent and preterm adult consultation groups, fathers expressed interest in statistics on neonatal conditions and complications, saying: ‘I wanted the stats,’ or: ‘I wanted numbers.’ They described a gendered division of parental labour, with mothers handling tasks ‘closer’ to the baby, such as expressing milk. These fathers regarded affinity for data as supporting their baby and combating their inability to fulfil stereotypical masculine ideals:

*‘They were telling us to stop looking at the machine and look at the baby. I couldn’t help [it]*. *I stood with the machine, I was analysing numbers… As a man, you want to try and protect them. You want… to try and do something, and it’s completely useless’* (Dad).

#### Bringing the data to life

Several participants desired to have individual case stories that represented their experiences; for them, the scale of the data muted how they related to the experiences of other families:

*‘You really need to provide actual stories of patients and parents, so that those statistics are not [just] statistics. So when you click on some of those dots, they take you to parent voices, interviews – good and bad’* (Preterm Adult Woman).

#### Yearning for predictions

Many participants expressed a need for, and limited access to, clear statements explaining risks, in a format such as: ‘X out of 10 000 preterm babies will experience this.’ They recognised each baby’s outcomes would be ‘different’, complicating prediction. However, they believed accessing large-scale data visualisations could help alleviate this:

*‘I asked for kind of stats because I needed to prepare myself… I had four days… so I think being armed with some form of stats [would have been helpful] ’* (Dad).

Although the tools were not designed to predict outcomes, one parent perceived data—specifically probability data—as being particularly helpful in navigating the swiftly changing risks that neonatal babies face:

*‘You think “we’re breezing this”… then it really hits the fan… [data] gives you something to hold onto… it allows you to manage your expectations… [the consultants’] knowledge isn’t going to be anywhere near as broad as the data… the conversation I had with them all the time was, “ what are the chances of X?” “ I don’t know, all babies are different.” “Okay but if you’ve got 10 k of these babies, what are the chances?” “We haven’t got that information”’* (Dad).

Parents pointed out that accessing the tool would offer an opportunity to reduce encroaching on clinicians’ time with requests for detailed explanations.

### Data managers, clinicians and network managers

The data managers, clinicians and network managers were recruited from the same neonatal network, and some regularly worked closely; therefore, the five themes which emerged from their consultations are reported together. The themes were comparativeness of data across units, additional outcomes and transfers, socioeconomic status and ethnicity, timeliness of data updates and widening availability of the tool.

#### Comparativeness of data across units

The most salient theme for this group was to understand differences between their unit’s outcomes and those of other comparable units:

*‘Something that allows us to make sure that we’re comparing like for like and that we’re not comparing a cohort of babies in… [our network] and thinking we’re doing terribly because [another unit is] doing much better… [when] actually their data is different…’* (Data Manager).

Presently, units define their standards by routinely monitoring data and comparing themselves to trends set by the collective work of units, but this can be challenging:

*‘I can pull things out from [electronic patient record system] but it might be very complicated… Rather than me having to constantly monitor data… if every year, I’m going to look at these lines illustrating differences across units, I know where I am heading to and where I need to work’* (Data Manager).

*‘Allowing us to identify where those differences in practice may be occurring, and pointers as to where we can go to either to review ourselves compared to peers around the country or which units we need to… focus on’* (Data Manager).

This group also focused heavily on the opportunities the tool would offer such as direct comparisons with other units. They recommended these be made between overarching operational delivery networks (ODNs) and between individual units within an ODN. One network manager reported that they were happy to feed back to their wider network, and expressed interest in sharing public-facing network-level data if anonymised and password protected:

*‘…I can see this taking off, it’s very detailed and it provides a lot of flexibility… there’s room for it to grow and develop… there’s lots of things like audit and parent engagement, I can imagine they’d like to see this data to kind of reassure them; it covers all the bases’* (Network Manager).

One network manager discussed specific ODN needs:

*‘It will certainly aid the standardisation of review… the ODNs all have a data manager, but there’s variance… each staff member is very different, and the needs of networks are quite different: it’s a genuine comparison with like for like… a huge step forward’* (Network Manager).

Another agreed, saying:

*‘Benchmarking is one of the key things we’ve struggled with for such a long time. Locally, we can compare apples with apples but my data and other managers’ would be apples and pears. That’s where the tools come into their own [sic]’* (Network Manager).

#### Additional outcomes and transfers

Discussion of additional outcomes beyond survival and length of stay focused on understanding reasons for admission and its variation between units. However, participants also expressed the desire for data on a wide range of outcomes, including breast feeding, intraventricular haemorrhage, modifiable factors, tube feeding in hospital and on discharge, probiotics, necrotising enterocolitis, lung disease, ventilator use and transfers. Transfers were also discussed as a particularly difficult phenomenon to track because of the varying reasons for why they occur:

*‘…I know there are two units whose babies should be coming to me – if they’re not, why? We don’t capture that… so we have to rely on audits…. It’s a big exercise capturing that, and the focus nationally is on trying to reduce transfers. What is their trajectory?’* (Clinician).

#### Socioeconomic status and ethnicity

Central to comparing units and ODN was identifying areas of disproportionate need, particularly how socioeconomic status and ethnicity shape their neonatal unit populations and admissions:

*‘…Some of the sliders in the tool would filter and say, “Out of your population of your 24–26 weekers, view that by ethnicity or deprivation”… It can help us understand where the maternity drivers need to be and to help some of those mums in a more focused and targeted way to understand getting better antenatal care…’* (Clinician).

#### Timeliness of data updates

Updating neonatal data was an integral complexity, caused by the length of time before results of clinical practice adjustments were observable:

*‘ If we made a change, we’re not going to see it for another year…. If we’ve been doing the wrong thing, it takes us a long time to find out’* (Data Manager).

Participants said the visualisation tool could provide ‘standardisation’ of and ‘more frequent access to’ data on a regular basis, but neonatal care processes cause a ‘lag’ in data access:

*‘Anything we do monthly we’re not going to have any outcomes unless they died within a few days. We don’t know lengths of stay for 3 or 4 months in some cases… Having it too quickly is detrimental, we don’t get the true picture…’* (Data Manager).

One participant emphasised the solution would lie in ‘access to timely data but not too timely data.’

#### Widening availability of the tool

One clinician described themself as a ‘technical dinosaur’ and, like the parent and preterm adult groups, said the visual nature of the tool was helpful. They also emphasised the importance of sharing visualisations with parents as PDF printouts and expressed strong interest in distributing the tool demonstration to data manager groups. They added that the inclusion of explanatory notes or supplementary material within the tool instead of simplifying existing content could support future interpretation. The network managers did not anticipate parental concerns regarding data use:

*‘ …They trust that professionals are doing that judgement call. If it’s good valid data, that’s why they’re showing them; as opposed to parents questioning data, then questioning what the answers are…’* (Network Manager).

The only exception was where there were very low numbers, for example, rare congenital anomalies, which could, coupled with geographical location or gestational age, identify babies. They felt confident they could reassure parents that data were insufficiently fine-grained for this.

### Researchers (trialists and epidemiologists)

A single theme emerged from the consultations with researchers: interrogating the data in the tool.

#### Interrogating the data

Trialists and epidemiologists enquired about the tool’s versatility, praising its usefulness for grants and study planning. They asked whether data will be broken down by conditions, and if capability exists for cross tabulations of more than two variables, such as filters exploring birth weight, gestational age and status 1 year later. One said:

*‘That would be really helpful for planning any study, not just trials, to get down to that level’* (Epidemiologist).

Trialists, like network managers, advocated for supplementary information including a data dictionary, suggesting this could be ‘hover and click.’ Like data managers, they cautioned against fine-grained attributes, explaining that, for example, the small number of 500 g birthweight babies *‘*etch themselves on your brain.*’* Another triallist expressed desire for data categories, ‘given the data spans ten years*’* (Triallist).

Another broadened this point, emphasising the inclusion of missing data in categories:

*‘It is still useful to have categories where there is a lot of missingness, even if they are not usable in a trial, as an indication of what is not currently routinely collected’* (Triallist).

## Discussion

We conducted consultations with 24 individuals from seven different stakeholder groups: parents, preterm adults, data managers, clinicians, network managers, trialists and epidemiologists. We identified four themes from parents and preterm adults: struggling to consume information, Dads and data, bringing the data to life and yearning for predictions; five themes from data managers, clinicians and network managers: comparativeness of data across units, additional outcomes and transfers, socioeconomic background and ethnicity, timeliness of data updates and widening the tool’s availability; and one theme from trialists and epidemiologists: interrogating the data.

PPPI on health data typically focuses on artificial intelligence,^23^ patient portals^24^ and decision-making tools^25^ and recommend problem solving by understanding the relationship between data capabilities and the role of patients and the public.^23–26^ Few involvement activities have focused specifically on providing public-facing data visualisations that are also useful to researchers, data professionals and clinicians. Our work contributes to the literature by consulting with all relevant stakeholder groups and synthesising their contributions. This enabled us to create and refine a tool which is useful to all groups, for a range of purposes specific to each. While other studies report that an artificial intelligence tool generated concerns,^23^ our stakeholders reported no concerns but were unanimously supportive and enthusiastic, citing visualisation functions as its greatest strength. Stakeholders had almost no criticisms of the tool, instead recognising its potential and desiring additional features. Parents saw the tool as an opportunity to inform themselves without burdening clinical staff with questions, while clinicians welcomed data visualisation as an aid to explaining potential outcomes to parents. All stakeholder groups engaged strongly with the tool’s potential and sought additional features. This suggests a universal need among the public, research and academic communities for more ways to address the broad, challenging and anxiety-inducing uncertainties in neonatal care.

The strength of this study was the inclusion of all seven relevant stakeholder groups and the very high level of consensus they reached. Study limitations are that participants were a self-selecting convenience sample, all were English speaking and a large group came from the same network, which may mean they are not representative of the wider population of stakeholders. Other limitations included the disproportionate gender balance in favour of mothers in the parent and preterm adult focus groups, and the potential for the limited number of fathers to have disproportionately affected the results. However, given that there is an underrepresentation of fathers in research involvement, any findings that such a consultation can generate are helpful.

We recommend that future health data involvement and engagement work should involve all stakeholders to form a full picture of perspectives and requirements. We intend to investigate the possibility of embedding parent and preterm adult narratives in the Neonatal Health Intelligence Tool alongside the statistical data, to enrich and humanise the experiences embodied by the data.

## Conclusions

All consultation participants united behind a need for the tool, praised its content and format, and desired additional features. Our consultations and qualitative inquiry with all groups of key stakeholders has enabled us to ensure the tool’s relevance and value to the communities it serves.

## Data Availability

Data are available on reasonable request.
