# Supplementary material for: Involving multiple stakeholders in assessing and reviewing a novel data visualisation tool for a national neonatal data asset
Source: BMJ Health Care Inform. 2023 Jan 31;30(1):e100694. doi: 10.1136/bmjhci-2022-100694 (PMC9890751; doi:10.1136/bmjhci-2022-100694)
Supplement: Supplementary data [file bmjhci-2022-100694supp001.pdf]

# SUPPLEMENTARY FILE 1

## Appendix 1 – Focus Group Topic Guides

### Parents, Patients, and Public Questions

- Would you use this tool? Would it be valuable to you? Why?
    - Right now the variables/outcomes are extremely limited, are these two things valuable for you?
- How do you feel about national aggregation of this data, or collection of neonatal data like this on a national scale? What would
- Was there any mention of large data like this during your experiences in the NICU? During your parents' experiences?
- Do these tools look reasonably easy to use?
- When/how do you think you would use them, if at all?
- If you wouldn't use them at all, what things would you like to see in them?
- What things would make you use them?

### Professionals Questions

- Would you use this tool? Would it be valuable to you?
    - Right now the variables/outcomes are extremely limited, are these two things valuable for you?
- Do these tools seem reasonably easy to use? What would make them easier to use?
- When/how do you think you would use them, if at all?
- If you wouldn't use them at all, what things would you like to see in them?
- What sorts of things would make you use them?
- What would you absolutely want tools like this for?
- What are your current processes like for examining and manipulating data in these ways? How do these tools compare?
